# Variation in recruitment across sites in a consent-based clinical data registry: lessons from the Canadian Stroke Network

**DOI:** 10.1186/1472-6939-7-6

**Published:** 2006-05-23

**Authors:** Donald J Willison, Moira K Kapral, Pierrot Peladeau, Janice A Richards, Jiming Fang, Frank L Silver

**Affiliations:** 1 Centre for Evaluation of Medicines, St. Joseph's Healthcare, Department of Clinical Epidemiology & Biostatistics, McMaster University Hamilton, Ontario, Canada; 2 Department of Medicine, University of Toronto, Institute for Clinical Evaluative Sciences, Division of General Internal Medicine and Clinical Epidemiology, University Health Network, Toronto, Ontario, Canada; 3 Centre for Bioethics, Clinical Research Institute of Montreal (IRCM), Centre francophone d'informatisation des organisations (CEFRIO), Montreal, Quebec, Canada; 4 Institute for Clinical Evaluative Sciences, Toronto, Ontario, Canada; 5 Division of Neurology, Department of Medicine, University of Toronto, Toronto, Ontario, Canada

## Abstract

**Background:**

In earlier work, we found important selection biases when we tried to obtain consent for participation in a national stroke registry. Recognizing that not all registries will be exempt from requiring consent for participation, we examine here in greater depth the reasons for the poor accrual of patients from a systems perspective with a view to obtaining as representative sample as possible.

**Methods:**

We determined the percent of eligible patients who were approached to participate and, among those approached, the percent who actually consented to participate. In addition we examined the reasons why people were not approached or did not consent and the variation across sites in the percent of patients approached and consented. We also considered site variation in restrictions on the accrual and data collection process imposed by either the local research ethics board or the hospital.

**Results:**

Seventy percent of stroke patients were approached, with wide variations in approach rates across sites (from: 41% to 86%), and considerable inter-site variation in hospital policies governing patient accrual. Chief reasons for not approaching were discharge or death before being approached for consent. Seventeen percent of those approached refused to participate (range: 5% to 75%). Finally, 11% of those approached did not participate due to language or communication difficulties.

**Conclusion:**

We found wide variation in approach and agree rates across sites that were accounted for, in part, by different approaches to accrual and idiosyncratic policies of the hospitals. This wide variation in approach and agree rates raises important challenges for research ethics boards and data protection authorities in determining when to waive consent requirements, when to press for increased quality control, when to permit local adaptation of the consent process, and when to permit alternatives to individual express consent. We offer several suggestions for those registries that require consent for participation.

## Background

The need for individual consent for the secondary analysis of existing data or for the use of data in clinical registries for a broad, long-range research agenda is highly contentious. Some researchers have called for a waiver of consent requirements for minimal risk research, arguing that obtaining individual consent would be impracticable and allowing individuals to opt-out would introduce bias into analyses [1-5]. Others, however, warn of the blurring of the distinction between research and clinical care, continual expansion of secondary uses of data for non-clinical purposes, and pressures to weaken human subject protections [6]. The responsibility of weighing the competing demands of scientific rigour and the protection of human subjects' rights falls squarely upon research ethics boards (REBs). In examining how to minimize analytic bias, waiver of consent is not the only option. In particular, REBs may first wish to consider what efforts have been taken to ensure quality control in the recruitment process.

Earlier, we reported selection biases associated with attempts to obtain consent for participation in a consent-based acute stroke registry, the Registry of the Canadian Stroke Network [5]. However, we also noted significant variation in approach and consent rates across sites, suggesting possible recruitment process issues that deserve attention. In this paper, we examine more closely the variation in recruitment across sites, and attempt to understand the reasons for variations in patient approach and agree rates. We do this to assist others who will be developing consent-based registries, in obtaining a sample that is as representative as possible of the larger population.

## Methods

The Canadian Stroke Network (CSN) is a collaborative effort of academic researchers, government, industry, and the non-profit sector, dedicated to decreasing the physical, social and economic consequences of stroke on the individual and on society. The Registry of the Canadian Stroke Network (RCSN) is a clinical database of patients with acute stroke patients seen at selected acute care hospitals across Canada. In this paper, we focus on "Phase 2" of the RCSN which took place between June 2002 and December  2002. Patients were recruited into the registry by experienced research nurses. Data collected included information about patient demographics and clinical symptoms, their hospital encounter, and quality of life and functional status (through a follow-up telephone interview).

We determined the percent of potentially eligible patients who were approached to participate in the Registry. We also examined what percent of those who were approached to participate actually consented to participate. Nurse-coordinators maintained a log documenting whether non-participation was due to patient refusal, inability to consent due to language or another communication barrier, or inability to approach the patient due to early discharge or other factors. In addition, we examined the variation across sites in the percent of patients who were approached to participate in the Registry and, of those, the percent who agreed to participate. Finally, we summarized the barriers encountered at individual sites through a survey of site coordinators.

## Results

### Reasons patients were not accrued into the Registry (Figure 1)

**Figure 1:**
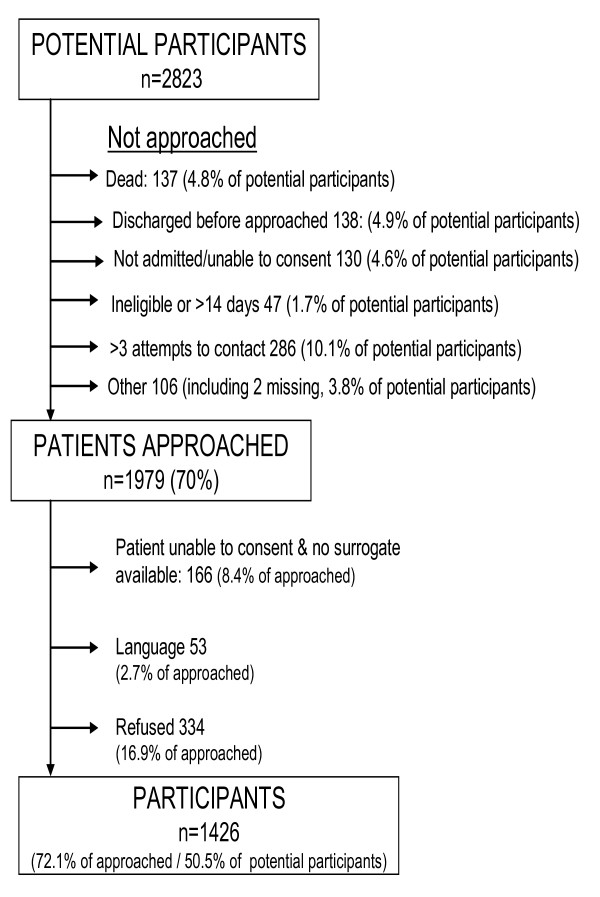
Patient accrual process – phase 2.

Overall, 70% of potential participants were approached and 72% of these were enrolled in the Registry, with an overall accrual rate of 50.5%. Logistical challenges in approaching patients accounted for 60% of non-accrual. Major reasons included: death before the patient could be approached (10%); discharge from hospital before being approached (10%); and inability to make contact with the patient or surrogate after more than 3 attempts (20%).

Of the patients approached, approximately 17% refused to participate. This is approximately 2.5 times greater than that encountered in pilot work (unpublished).

Eight percent of patients were unable to consent due to communication difficulties with no surrogate available. Another 3% of those approached were not administered the consent form because their mother tongue (and that of their surrogate) was not English or French.

### Variation in approach, agree, and overall participation rates (Figure 2)

**Figure 2:**
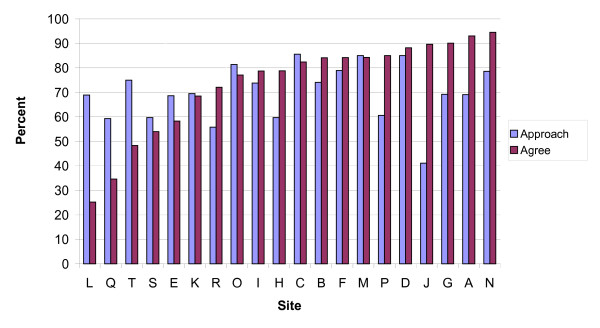
Approach and agree rates by site: sorted by agree rate.

The percent of eligible patients approached varied across sites from 41% to 86%, with a mean of 70%. The agree rate averaged 74% of those approached (lowest 25%, highest 95%). Approach and consent rates were not correlated across sites.

### Supports and constraints on data collection (Table 1)

**Table 1:** Supports and barriers to patient accrual and mean approach rates

		**Mean approach rate (n)**		**t-test p value**
		
		**Sites answering "Yes"**	**Sites answering "No"**	
Supports Available for the Registry	1. Do you work with the "Stroke Team" (attend ward rounds, discharge planning meetings, receive referrals or patient lists from the team, etc.)	70.4 (11)	69.4 (9)	0.8651
	2. Do your physicians (attending/investigator/residents/fellows) help you obtain patient consents?	73.0 (9)	71.6 (9)	0.7503
	3. Is there a neurology/stroke prevention clinic where TIA/stroke patients are seen following emergency department visits at your hospital?	68.9 (15)	73.0 (5)	0.5084
	4. Do you have support from your emergency department (notification of new patients, providing brochures for patients not admitted to hospital, etc.)	80.5 (5)	66.9 (14)	0.0183
Barriers to Recruitment	1. Will your institution allow you to collect the minimal dataset on all patients?	70.1 (18)	69.0 (2)	0.901
	2. Will your institution allow you to obtain lists of potential stroke patients from ED and the wards?	70.4 (19)	60.6 (1)	0.417
	3. Will your institution allow you to directly approach admitted patients to consent to participate in the Registry?	71.1 (18)	59.9 (2)	0.1962
	4. If a "potential" registry participant has been discharged (or never admitted) will your institution allow you to contact the patient to participate in the registry?	72.0 (13)	65.7 (6)	0.2877
	5. Will your institution allow you to obtain telephone consent?	69.2 (6)	69.7 (12)	0.9446

There was considerable variation across sites in supports for and constraints on approaching patients. Only five sites (25%) had active support from the emergency department (e.g. notification of new patients, providing brochures for patients not admitted to hospital). These sites achieved a substantially higher approach rate than did the sites without such support (average 80.5% vs. 67%; p < 0.02).

We found approach rates to be lower:

• When lists of potential stroke patients could not be obtained from the emergency department or the wards (60.6% vs. 70.4%);

• When coordinators could not approach patients directly. In these cases, the physician responsible for care had to first approach the patient. (59.9% vs. 71.1%); and

• When coordinators could not make contact with the patients after they had left the hospital (65.7% vs. 72.0%).

None of these results was statistically significant, although this may have been due to inadequate statistical power. Approach rates were no different in sites where the local principal investigator actively participated in the recruitment and where the nurse recruiter worked closely with the stroke team.

## Discussion

We found wide variation across sites in both the rate at which potentially eligible patients were approached to participate in the Registry and in consent rate. Based on our discussions with study coordinators, we learned that some of the difference in approach rates was due to variations in the interpretation of provincial data protection laws, and by site-specific limitations imposed by hospitals on the conditions under which patients could be approached. In some cases, the restrictions applied by hospital administration went over and above those applied by the research ethics boards or by provincial laws. In addition, sites receiving support from their emergency department (e.g. notification of new patients, providing brochures for patients not admitted to hospital) had substantially higher approach rates.

We observed an overall refusal rate of 17% with wide variation across sites (5% to 75%). This indicates unevenness in the approach to recruitment across sites. Ideally, it would be helpful to ask those who refused why they refused. This information is not available from the Registry. However, in future research projects, where consent is required, it would be instructive to learn why people refuse to participate, so as to be responsive to concerns raised.

### Regulatory and governance context

Canadian federal and provincial data protection laws allow for exemptions from consent for research purposes where, among other conditions: (a) the research cannot be achieved without using personal information, and (b) it is impracticable to obtain consent [7]. However, none of the provinces' legislation provides clear guidance as to the circumstances under which obtaining consent may be deemed impracticable. In Alberta, Saskatchewan, and Ontario, legislation specifically identifies this to be the purview of the research ethics board (REBs) [8].

Article 3.4 of the Tri-Council Policy Statement (TCPS) – the document that articulates the standards in Canada for REBs of institutions receiving funding from any of the three major federal granting councils – states that the "REB ***may***[*our emphasis*] also require that a researcher's access to secondary use of data involving identifying information be dependent on: (a) the informed consent of those who contributed data or of authorized third parties;..." [9]. No specific guidance is provided as to the criteria for determining whether or not consent should be required for secondary use of existing personal information for research. However, two of the fundamental guiding ethical principles articulated in the TCPS are respect for free and informed consent and respect for privacy and confidentiality. Therefore, to be consistent with the values, purpose, and protections advanced in the TCPS, the onus for demonstrating a reasonable exception to the requirement for consent should fall on the researcher.

In 2005, the Canadian Institutes of Health Research (CIHR) issued its Best Practices for Protecting Privacy in Health Research[8]. Element 3 of the document includes detailed guidance as to the factors to consider when determining whether or not a research project should receive exemption from consent for secondary use of personal information. One of the provisions is quite broad – if, due to the size of the population, the proportion likely to have relocated or died, or lack of continuing relationship with the data holder:

"...there is a risk of introducing bias into the research because of the loss of data from segments of the population that cannot be contacted to seek their consent, thereby affecting the validity of results and/or defeating the purpose of the study."

We recognize the risk that this provision runs the risk of becoming a "trump card" over privacy concerns. Accordingly, it places a heavy onus on REBs to determine when to allow use of the data without consent and when to press for increased quality control in recruitment. This is relatively simple when it has been demonstrated that the vast majority of the potential research participants would be willing to allow their information to be used. It is much more difficult when, as we found, a non-trivial proportion of people approached refuses to participate.

Section 39 of the 2004 Ontario Personal Health Information Protection Act permits the disclosure of personal health information without consent to "prescribed registries" for the purpose of statistical analysis [12]. A handful of registries, including the Registry of the Canadian Stroke Network, are among the prescribed registries [10].

### Experience elsewhere

The limited published literature on recruitment suggests that challenges in variation in recruitment faced by the Registry of the CSN are not unique. While researchers associated with the Mayo Clinic in Minnesota were able to achieve consent rates in excess of 95% to participate in a broad cross-section of disease-registries, there was variation both across sites and by diagnosis [11,12].

It appears, though, that consent-seeking alone is not exclusively responsible for incomplete accrual. Across 91 U.K. clinical databases listed under the Directory of Clinical Databases (DoCDat), completeness of patient recruitment appears to be similar for databases that do and do not require individual consent for enrolment [13].

In a different context, Gross and colleagues examined patient accrual for 172 clinical trials in four high-impact medical journals. They found very poor reporting of the patient accrual process, with only 31 studies (18%) screening from a consecutive series of patients [14].

### Lessons learned

Many researchers will still need to obtain informed consent for patient participation in their registry projects – for example, where there will be direct patient contact, where genetic information will be included or linked, or in particularly stigmatizing medical conditions. Several lessons can be learned from our experience with developing a consent-based registry. These lessons are derived from the data presented in this paper and from our discussions with site coordinators and co-investigators:

(1) The consent process needs to be thoroughly pilot tested under 'real-world conditions' with gradual roll-out to participating sites. One should anticipate ample lead time to develop, test, and implement the entire concept – particularly the consenting process and staff responsibilities.

(2) Close communications need to be established early and maintained with research ethics boards and health care institutions. This is probably best accomplished through a single contact-person working with each REB and hospital from the outset of the project.

(3) Accountability requirements for those responsible for obtaining consent should be as consistent as possible. Nurse coordinators in this study had a dual accountability: to the central coordinator and to local site principal investigators.

(4) Consider staging the implementation process, so as to build on the successes of the less complicated recruitment scenarios. For example, from the outset, we tried to recruit patients with transient ischemic attacks. This was ambitious, as these particular patients usually were not admitted to the hospital, and they constituted a large proportion of our patients not approached.

(5) Use a multi-pronged strategy for recruitment when potential registry participants have multiple points of access or care trajectories (e.g. both inpatient and outpatient treatment). Obtaining consent may be more feasible when repeated outpatient visits allow increased contact and trust.

(6) Obtain firm support of those departments that have first contact with target patients (e.g. Emergency) to identify potential participants and provide them with information and support to implement screening processes.

(7) Consider random sampling strategies to reduce workload, rather than including all consecutive patients. This was a strategy we implemented in Phase 2 in institutions with particularly high volumes of stroke patients. We found this increased the approach rate.

(8) Ongoing monitoring and feedback on accrual help to increase and sustain higher accrual rates and interest.

(9) Consent forms in other languages and access to translators may be required for projects operating in jurisdictions with multi-ethnic populations. Usually, such hospitals have a roster of translators for such situations.

(10) Elicit ongoing patient feedback – particularly from those who hesitate or refuse to participate – to ascertain what concerns they may have. While some refusers may not wish to share this information, if this is done in a way that does not pressure patients, then it can provide valuable feedback.

### Long-run changes are needed

Concern has been expressed elsewhere that, for multi-centered studies, the process of research ethics approval is very time consuming, with considerable duplication of effort and local idiosyncratic restrictions that offer little perceived gain [15-17]. In some countries, a centralized review process has been implemented for such multi-centered studies. While intended to streamline the review process, in some cases this has simply added another level of bureaucracy [18]. Even greater standardization of the process would be helpful. Assimilation of the CIHR privacy guidelines into the review process could help to harmonize the interpretation of acceptable recruitment practice.

In our study, the chief source of variation in administrative requirements came not from the REBs but from the data stewards – acute care hospitals. In particular, we found major differences in (a) ability to coordinate with the emergency department in the recruitment process; and (b) hospital policy as to whether, and at what point, potential registry participants could be approached to participate. In part, this can be resolved through education of health care institutions as to what is permitted by the law.

Looking forward, numerous registry and linked electronic health record data collection activities are being planned in North America and Europe. Perhaps it is time to re-think how we go about recruiting patients into these registries [19]. For example, would it be more efficient to shift responsibility for patient accrual from managers of individual projects to the institution and for a network of institutions to develop common protocols across institutions for obtaining consent for use of clinical records, obtaining biological samples, follow-up surveys, and linkage of clinical data with administrative records?

## Conclusion

We have described numerous challenges in developing and implementing a consent-based registry for stroke patients. We believe that ours is not a unique experience. Our attempts have led to important sampling biases that limit the generalizability of our data. We have also demonstrated important quality control issues in conducting a multi-centered registry. The teasing out of these issues represents a major challenge to research ethics boards and data protection authorities who are charged with the responsibility of determining when to allow collection of information without consent, when to press for increased quality control in recruitment, and when to permit local adaptation of the recruitment process. We hope that the experience of the Registry of the Canadian Stroke Network will contribute to future-oriented solutions.

## Competing interests

All the authors except Peladeau have been sponsored through the Canadian Stroke Network to attend one or more annual meetings of the Canadian Stroke Network or related conferences. The authors have no other financial or non-financial competing interests.

## Authors' contributions

DW conceived the study, drafted the manuscript, and produced subsequent revisions. Remaining authors reviewed and revised the manuscript. JF performed the statistical analyses. JR coordinated the data collection. All authors read and contributed to successive drafts of the manuscript.

## Pre-publication history

The pre-publication history for this paper can be accessed here:


